# 

**Published:** 1828-01

**Authors:** 


### THE LONDON MEDICAL AND PHYSICAL JOURNAL.

EDITED BY

RODERICK MACLEOD, M.D.

PHYSICIAN TO THE
WESTMINSTER GENERAL DISPENSARY,
AND LECTURER
ON THE THEORY AND PRACTICE OF PHYSIC, AND ON MATERIA MEDICA,
AT THE SCHOOL IN GREAT WINDMILL STREET.

(VOL. LIX.)

NEW SERIES, VOL. IV.

Et quoniam variant morbi, variabimus artes;

Mille mali species, mille salutis erunt.

Surgeon Gen'l's Office LIBRARY Washington, D.C.

45592

LONDON:

JOHN SOUTER, 73, ST. PAUL'S CHURCH-YARD,

AND TO BE HAD OF ALL THE MEDICAL BOOKSELLERS.

1828.

W1
LO31

*J. and C. Adlard, Printers,*

*Bartholomew Close.*

### MONTHLY LIST OF MEDICAL BOOKS.

[*No Books can be entered on this List except a copy be sent for the purpose; the titles of works having frequently been transmitted to us, as published, which have not appeared for weeks, or even months, after.*]

Observations on the Properties and Effects of the expressed Oil of the Seed of Croton Tiglium; together with the Botanical History, and a correct coloured Engraving, of the Plant. By JOHN FROST, F.S.A. F.L.S. F.H.S. &c. —8vo. London, 1827.

A Manual of Surgical Anatomy, by H. M. EDWARDS, D.M.P. Translated, with Notes, by WILLIAM COULSON, Demonstrator of Anatomy at the Medical School, Aldersgate-street.—12mo. London, 1827.

An Essay on Gout: in which its actual Predisponent, Proximate, and Exciting Causes are clearly defined; and its Preventive and Curative Indications fully demonstrated, upon new Pathological Principles. By P. P. P. MYDDELTON, M.D. &c.—8vo. Fourth Edition. Bath, 1827.

A Preliminary Dissertation, illustrative of a new System of Pulmonary Pathology, supported by a Series of conclusive Physiological Experiments: combining a rational Theory with a successful Method of conducting the Cure of Consumption. By P. P. P. MYDDELTON, M.D. &c.—8vo. Bath, 1825.

### METEOROLOGICAL JOURNAL,

### NOTICES.

*We have received the Communications of* Mr. LYONS, Mr. GORE, Mr FERGUSSON, *and* Mr. SELWYN.

Mr. G. *will perceive that we received the volumes.*

